# Development and validation of a hierarchical approach for lymphoma classification using immunohistochemical markers

**DOI:** 10.1002/cam4.70120

**Published:** 2024-10-23

**Authors:** Jiming Xu, Yunfei Shi, Mengxuan Cui, Yao Wang, Wenhui Fan, Jingping Yun, Linfeng Li, Muyan Cai

**Affiliations:** ^1^ Department of Automation Tsinghua University Beijing China; ^2^ Yidu Cloud Technology Inc Beijing China; ^3^ Key Laboratory of Carcinogenesis and Translational Research, Department of Pathology, Ministry of Education Peking University Cancer Hospital and Institute Beijing China; ^4^ Collaborative Innovation Center for Cancer Medicine, State Key Laboratory of Oncology in South China, Department of Pathology Sun Yat‐sen University Cancer Center Guangzhou China

**Keywords:** diagnosis, immunohistochemistry, lymphomas, machine learning

## Abstract

**Background:**

Accurate lymphoma classification is critical for effective treatment and immunohistochemistry is a cost‐effective and time‐saving approach. Although several machine learning algorithms showed effective results, they focused on a specific task of classification but not the whole classification workflow, thus impractical to be applied in clinical settings. Thus, we aim to develop an effective and economic machine learning‐assisted system that can streamline the lymphoma differential diagnostic workflow using EBER in situ hybridization and immunohistochemical markers.

**Methods:**

We included pathological reports diagnosed as lymphomas from two cancer centers (Sun Yat‐sen University Cancer Center and Peking University Cancer Hospital & Institute). We proposed a hierarchical approach that mimicked the human diagnostic process and employed simplified panels of markers to perform a series of interpretable classification. The diagnostic accuracy for lymphoma pathological subtypes and the markers saving ratio were investigated in both temporal independent population and external medical center.

**Results:**

A total of 14,927 patients and corresponding immunohistochemical results from two cancer centers were included. The proposed system had high discriminative ability for differentiating lymphoma pathological subtypes (measured by mean AUC in three validation cohorts, non‐Hodgkin and Hodgkin lymphoma: 0.959; non‐Hodgkin subtypes: 0.983; B‐lymphoma subtypes: 0.868; T‐lymphoma subtypes: 0.962; DLBCL subtypes: 0.957). In addition, the system's well selected characteristics can contribute to the development of agreement on panels of markers for differential diagnosis and help minimize cost of immunohistochemical marker techniques (measured by marker saving ratio compared to real clinical settings, internal primary‐stage cohort: 16.45% saved, *p* < 0.001; internal later‐stage cohort: 21.73% saved, *p* < 0.001; external cohort: 3.67% saved, *p* < 0.001).

**Conclusions:**

Machine learning‐based hierarchical system using EBER in situ hybridization and IHC markers was developed, which could streamline the workflow by sequentially determining each lymphoma pathological subtype. The proposed system proved to be effective and cost‐saving in independent and external validation, thus could be adopted affordably in future clinical practice.

## 1 INTRODUCTION

Lymphoma is a disease that, in general, requires specialized knowledge and experience to diagnose and categorize properly. Precise diagnosis, tailored treatment strategies, and better patient outcomes rely heavily on accurate lymphoma classification.^1^, ^2^ However, lymphoma classification can be challenging owing to the great variety of lymphoma subtypes.^1^, ^3^ Currently, the World Health Organization (WHO) classification system is the most widely used approach for categorizing lymphomas into subtypes based on their morphology, immuno‐phenotypes, and genetic characteristices.^4^

Many strategies for diagnosing lymphoma have been used in clinical practice. As compared to DNA‐ and RNA‐based approaches, immunohistochemistry (IHC) is a more cost‐effective and time‐saving technique for lymphoma differential diagnosis. IHC is utilized in the context of lymphoma classification to identify specific proteins or markers that are indicative of certain significant consequences for prognosis and therapy.^5^ This technique has been wildly used by pathologists. For example, the most frequent pan‐B‐cell marker is CD20, while the most common pan‐T‐cell antigen is CD3. Also, a number of significant studies on the use of IHC data to differentiate between distinct lymphoma variants have been reported.^6^, ^7^, ^8^

With the innovations of data science, many machine learning algorithms have been developed and extensively employed in the field of pathological recognition. IHC results have been utilized in several investigations, including the algorithms developed by Hans and Choi, to categorize diffuse large B‐cell lymphoma (DLBCL) subtypes.^6^, ^7^ There is strong evidence linking several of these findings to their corresponding gene expression profile (GEP). Unfortunately, most of these studies lacked a comprehensive diagnosis workflow in favor of focusing on a specific task of classification. To complete a series of interpretable categorization, a streamlined system is required that can automate the human‐like diagnostic process.

As no single antigen can be relied upon for definitively classifying lymphoma pathological subtypes, appropriate IHC marker panels were investigated as an alternative to single marker for the diagnosis of lymphoma. However, recommendations for marker panels are inconsistent in previous studies^6^, ^7^, ^8^, ^9^, ^10^; and few studies have explored whether or not these panels may be streamlined. There was also a discrepancy between the two centers in this study with regards to the marker panels observed in real‐world clinical settings, suggesting that the determination of marker panels may be influenced by local clinical practice as opposed to a universal concept. Thus, there is a need for lymphoma diagnostic panels that are independently verified and easy to use.

In this study, we aim to build a streamlined hierarchical system for lymphoma diagnosis, using machine learning approaches to provide simplified panels of indicators at various stages of the hierarchy. To implement this in clinical settings, we integrate this approach with an already existing machine that provides EBER in situ hybridization and IHC data in a stepwise manner. Our system comprises five modules: first, patients with lymphoma are classified into non‐Hodgkin lymphoma (NHL) and Hodgkin lymphoma (HL); second, specific variants of NHL (B‐cell lymphoma, T‐cell lymphoma, or NK‐cell lymphoma) are identified; third and fourth, B‐cell lymphoma and T‐cell lymphoma are classified into their variants; and finally, if identified as DLBCL, the specific cell‐of‐origin subtype of DLBCL, germinal center B cells (GCB) or non‐GCB, is classified.

## 2 MATERIALS AND METHODS

**FIGURE 1:**
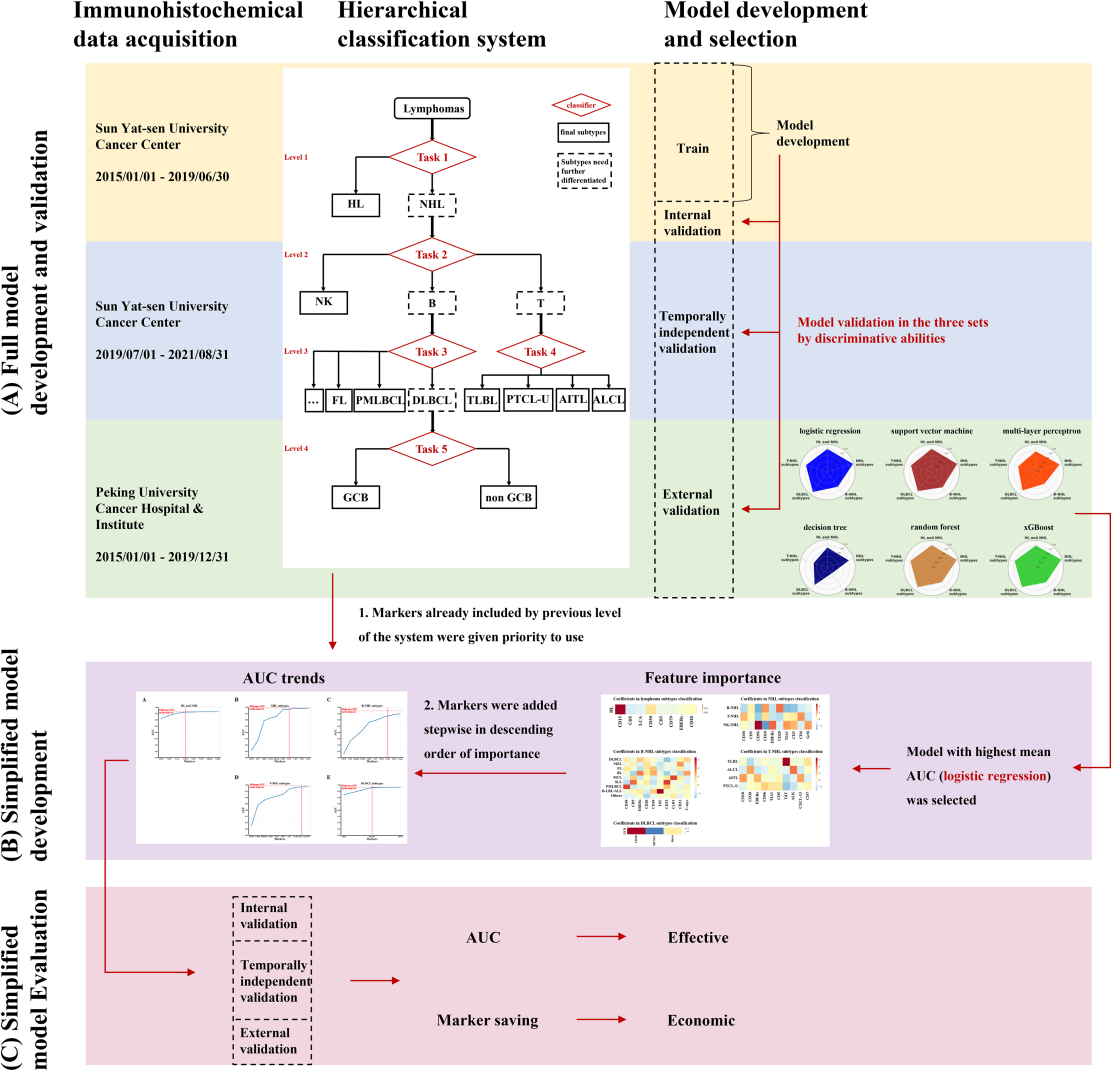
Workflow of the study. (A) Pathological reports diagnosed as lymphomas were retrospectively retrieved from two cancer centers, consisting of three independent sets. Pathological subtypes were labeled according to the hierarchy developed by WHO. Using full panel of markers, six machine learning models were developed and evaluated. (B) Based on the optimal machine learning algorithm that with highest mean AUC in the three validation sets, features were ranked by importance and selected to build five simplified models. (C) Simplified system was then evaluated in the three validation sets by its discriminative ability and economic effect. HL, Hodgkin lymphoma; NHL, non‐Hodgkin lymphoma; DLCBL, diffuse large B‐cell lymphoma; FL, follicular lymphoma; MZL, marginal zone lymphoma; MCL, mantle cell lymphoma; BL, Burkitt lymphoma; SLL, small lymphocytic lymphoma; PMLBCL, primary mediastinal (diffuse) large B‐cell lymphoma; B‐LBL/ALL, B lymphoblastic lymphoma/leukemia; Others, other B‐NHL subtypes; ALCL, anaplastic large cell lymphoma; AITL, angioimmunoblastic T‐cell lymphoma; TLBL, T lymphoblastic lymphoma; PTCL‐U, peripheral T‐cell lymphoma‐unspecified; GCB, germinal center B cells.

This is a multicenter observational study involved patients diagnosed as lymphomas at Sun Yat‐sen University Cancer Center and Peking University Cancer Hospital & Institute. Based on the 4th revised edition of the WHO classification of Hematopoietic Tumors (Figure S1), the pathological subtypes of these records were labeled. The workflow of the study is shown in Figure 1.

### 2.1 Participants

We included the pathological reports diagnosed as lymphomas at Sun Yat‐sen University Cancer Center and Peking University Cancer Hospital & Institute as the internal (from January 1st 2015 to August 31st 2021, *N* = 8808) and external cohorts (from January 1st 2015 to December 31st 2019, *N* = 6119), respectively, along with their corresponding EBER in situ hybridization and IHC results. Reports having multiple lymphoma diagnoses were eliminated. Through this refinement, the study exclusively encompasses individuals receiving their primary diagnosis, while excluding those experiencing disease recurrence. The internal cohort consisted of two temporally independent cohorts, the internal primary‐stage (from January 1, 2015 to June 30, 2019, *N* = 4263), which was randomly split into train and internal validation cohorts, and the internal later‐stage cohorts (from July 1, 2019 to August 31, 2021, *N* = 4545). The cohort sizes and predetermined markers based on medical knowledge are described in Table 1. Only reports that matched these basic conditions were used as valid data in each task's model training and validation. This approach ensured the reliability and accuracy of the data used for analysis.

**TABLE 1:** Task specific number of classes, minimum required markers, full panel of markers, and size of filtered datasets.

Task of classification	Number of Classes	Minimum required markers	Full panel of markers	Train set (*N*)	Internal validation set (*N*)	Temporally independent validation set (*N*)	External validation set (*N*)
HL and NHL differentiation	2	CD30	CD15, CD30, CD5, LCA, CD3, CD20, CD79, EBERs	1777	445	2528	2716
NHL subtypes differentiation	3	CD3 and CD20	CD56, CD20, EBERs, CD3, CD4, CD10, TIA1, MUM‐1, CD30, GrB, CD2, Perf, CD7, Pax‐5, CD79, CD8, CD21, CD5	2745	687	3746	5057
B‐cell lymphoma subtypes differentiation	9	CD20, CD10, CyD1, and (CD5 or Bcl‐2)	CyD1, TdT, CD23, CD30, CD20, EBERs, Bcl‐6, MUM‐1, CD138, CD10, CD21, CD38, CD5, CD43, Ki67, C‐myc, Pax‐5, Bcl‐2, ALK	1365	342	1688	2320
T‐cell lymphoma subtypes differentiation	4	CD3, or CD8, or CD30, or ALK	TdT, CXCL‐13, ALK, CD30, CD10, CD3, EBERs, CD56, GrB, CD4, TIA1, Ki67, Bcl‐6, CD2, CD7, CD43, CD5, Bcl‐2, CD8	306	77	372	425
DLBCL subtypes differentiation	2	CD10 and MUM‐1	CD10, MUM‐1, Bcl‐6	648	164	594	963

Abbreviations: DLCBL, diffuse large B‐cell lymphoma; HL, Hodgkin lymphoma; NHL, non‐Hodgkin lymphoma.

### 2.2 Measurements

The pathological subtypes were categorized by three experienced pathologists served as the ground truth. Several pathological images were included in Figures S2–S5. The EBER in situ hybridization and IHC markers from each included pathological report included in this investigation were employed as biomarkers in this analysis. The pattern‐based natural language processing (NLP) method was used to extract the name and value of markers. Researchers investigated the extraction accuracy and found it to be reliable.

Although hundreds of different markers were retrieved, most of them were not routinely used. To ensure the selection of the most relevant markers for each lymphoma classification task, experienced pathologists chose particular subsets of markers as candidate panels (Table 1).

### 2.3 Hierarchical classification system design

The development of classification system is shown in Figure 1. According to the 4th revised edition of the WHO classification of Hematopoietic Tumors, we developed a hierarchical system consisting of five steps for lymphoma classification (Figure S1). The first step is to differentiate NHL from HL for lymphoma patients. If identified as NHL in the first step, the second step is to identify of specific variants of NHL, including B‐cell lymphoma, T‐cell lymphoma, or NK‐cell lymphoma. The third and fourth steps are the classification of B‐cell lymphoma and T‐cell lymphoma, where a decision is made when the phenotypes reach the last step, which includes HL, NK/T‐cell lymphoma, all variants of B‐cell and T‐cell lymphoma except for DLBCL, GCB, or non‐GCB.

The first stage of development was to identify the optimal algorithm. Using full panel of markers, predictive models for the five steps were developed and evaluated (Figure 1A). Specifically, we generated six machine learning models using the train set for each classification task, employing algorithms such as logistic regression (LR), support vector machine (SVM), multi‐layer perceptron (MLP), decision tree (DT), random forest (RF), and extreme gradient boosting (xGBoost). Each marker for DT, RF, and xGBoost was encoded as a categorical feature (negative, positive or unknown). By using one‐hot encoding, each marker was transformed into two binary statuses (positive or negative) for LR, SVM, and MLP.

Performance was evaluated on the internal validation, temporally independent validation, and the external validation sets for each classification task. The algorithm with the best overall discriminative capability, as measured by sum of area under ROC curve (AUC) of all tasks on the three validation datasets, was selected as the best candidate algorithm. Using the best candidate algorithm, the model created by the best candidate algorithm for each task was dubbed the *Full Model*, as it leveraged full candidate marker panels for that task. The AUC of full model in the train set was then used as the *Reference AUC* for future simplification model for each task.

The second stage of development was to make the hierarchical classification system more practical and cost‐effective, so we implemented two steps to simplify the model and marker panels (Figure 1B). First, based on the optimal machine learning algorithm that with highest mean AUC in the three validation sets, we ranked markers by importance and added them to models in descending order of importance until the AUC achieved 99% (95% for the task of B‐cell lymphoma subtypes classification due to its higher difficulty) of the *Reference AUC* in the corresponding full model. We used the maximum coefficients of markers in LR to determine feature importance. Secondly, we prioritized markers that were previously included throughout the sequential process of hierarchical classification tasks. This means we utilized all tested markers before adding new markers to the panel. By doing this, we created *Simplified Panels*, which require minimum number of markers possible. The *Simplified Panels* were then used to train simpler models.

The third stage of development was to evaluate the simplified system by its discriminative ability and economic effect (Figure 1C). To evaluate the efficacy of the simplified panels, we assessed the performance of the five tasks using a variety of evaluation metrics include AUC (calculated using “one‐over‐rest” for multi‐class classification tasks), accuracy, macro‐precision, macro‐recall, and marco‐F1 on three validation sets. We also compared the number of markers in the *Simplified Panels* with the number evaluated in real‐world clinical settings to determine the extent of the potential savings.

### 2.4 Statistical analysis

Chi‐square test was employed to compare marker expressions among subtypes. Numbers of markers required by proposed system and actually tested in clinical settings were compared by *T* or Mann–Whitney *U*‐test, as appropriate. Two‐sided *p* values less than 0.05 were regarded as statistically significant. All analyses were conducted with Python 3.9.7.

## 3 RESULTS

### 3.1 Participants and markers

The train cohort (Table 1) consisted of 1777 genuine lymphoma cases for discriminating between HL and NHL, with 84.81% (*n* = 1507) of samples diagnosed as NHL. Among the 2745 cases used for NHL subtype classification in the train cohort, B‐cell lymphoma was the most common, constituting 77.67% (*n* = 2132) of all cases, while T‐cell lymphoma and NK‐cell lymphoma made up 13.30% (*n* = 365) and 9.03% (*n* = 248) of the subtype, respectively. With B‐cell lymphoma subtype, DLBCL was the most prevalent in the train cohort, accounting for 45.93% of all B‐cell lymphoma cases (*n* = 627 out of 1365). The prevalence of subtypes was similar across the other three datasets, and the percentages of subtypes and markers in all four cohorts are presented in Tables S1 (train set), S2 (internal validation set), S3 (temporal independent set), and S4 (external validation set) in Appendix S1.

### 3.2 Models from the whole candidate panel

Among the three validation sets, LR outperformed other models in the discriminative ability, with the greatest average AUC (Table S5 in Appendix S1). The mean AUCs in the internal validation, temporally independent validation, and external validation sets were 0.967, 0.961, and 0.958, respectively. As a result, the LR in the five tasks were selected as the *Full Models*, and the AUCs of the LR in the train set were used as *Reference AUCs* for future model simplification. For the tasks of HL versus NHL, NHL subtypes, B‐cell lymphoma subtypes, T‐cell lymphoma subtypes, and DLBCL subtypes classification, the *Reference AUCs* in the train set were 0.966, 0.995, 0.973, 0.993, and 0.983, respectively. It was easier to classify HL versus NHL, NHL subtypes, T‐cell lymphoma subtypes, and DLBCL subtypes. In the three validation sets, the above four tasks had mean AUCs of 0.958, 0.993, 0.960, and 0.964, respectively, whereas the task of B‐cell lymphoma subtypes was more difficult with lowest mean AUC of 0.934.

### 3.3 Hierarchical classification system simplification

**FIGURE 2:**
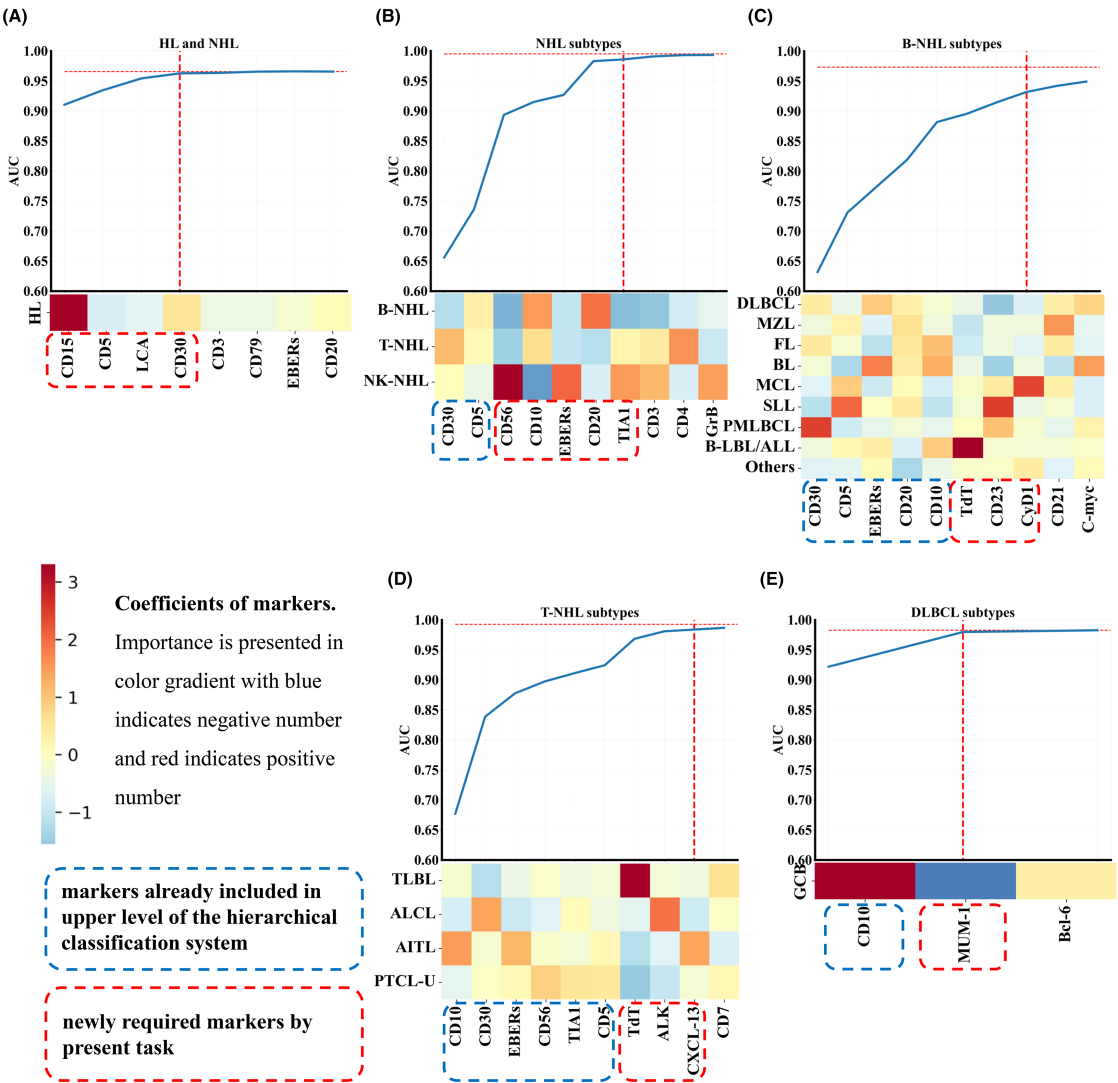
The AUC trends throughout the gradual addition of markers in the train set. For the tasks of (A) HL versus NHL, (B) NHL subtypes, (C) B‐cell lymphoma subtypes, (D) T‐cell lymphoma subtypes, and (E) DLBCL subtypes classification, the importance of the marker is presented in color gradient, with color blue indicates a negative number and color red indicates a positive number. First, markers already included by previous level of the system (Blue boxes) were given priority to use in model development. Then, markers were added stepwise in descending order of importance. Red boxes label extra markers required by this task to satisfy the requirements of AUCs. AITL, angioimmunoblastic T‐cell lymphoma; ALCL, anaplastic large cells lymphoma; BL, Burkitt lymphoma; B‐LBL/ALL, B lymphoblastic lymphoma/leukemia; DLCBL, diffuse large B‐cell lymphoma; FL, follicular lymphoma; GCB, germinal center B cells; HL, Hodgkin lymphoma; MCL, mantle cell lymphoma; MZL, marginal zone lymphoma; NHL, non‐Hodgkin lymphoma; Others, other B‐NHL subtypes; PMLBCL, primary mediastinal (diffuse) large B‐cell lymphoma; PTCL‐U, peripheral T cells lymphoma‐unspecified; SLL, small lymphocytic lymphoma; TLBL, T lymphoblasts lymphoma.

Using train data, we ranked importance of markers for each task by LR approach (Table S6 in Appendix S1) using absolute values of coefficients. We then simplified the models and opted on the simpler panel of markers using the train data. Figure 2 depicts the coefficients of markers and AUC trends throughout the gradual addition of markers by descending order of importance. When the AUC in the train set reached 99% (or 95% for the task of B‐cell lymphoma subtypes) or higher of reference AUC in the matching full model, a simpler model was generated for that task (Table S7 in Appendix S1).

### 3.4 Evaluation of the hierarchical classification system

We assessed the simplified hierarchy's discriminative ability (Table 2) as well as economic effectiveness (Table 3).

**TABLE 2:** Validation results of the simplified system. For each task of classification, simplified model was developed using identified panel of markers, and was evaluated in the three validation sets by a variety of metrics, include area under ROC curve (AUC), accuracy, macro‐precision, macro‐recall and marco‐F1, with AUC calculated using “one‐over‐rest” for multi‐class classification tasks. v1: internal validation; v2: temporal independent validation; v3: external validation.

Task of classification	Simplified panel of markers	Validation set	AUC of full model	AUC	Accuracy	Macro‐F1	Macro‐precision	Macro‐recall	Weighted‐average‐F1	Weighted‐average‐precision	Weighted‐average‐recall
HL and NHL differentiation	CD15, CD5, LCA, CD30	Internal validation	0.960	0.961	0.935	0.865	0.895	0.841	0.932	0.932	0.935
		Temporally independent validation	0.956	0.956	0.936	0.860	0.865	0.856	0.936	0.935	0.936
		External validation	0.960	0.961	0.911	0.880	0.886	0.874	0.911	0.910	0.911
NHL subtypes differentiation	CD30, CD5, CD56, CD10, EBERs, CD20, TIA1	Internal validation	0.993	0.980	0.939	0.883	0.890	0.876	0.938	0.937	0.939
		Temporally independent validation	0.993	0.983	0.934	0.874	0.900	0.851	0.932	0.931	0.934
		External validation	0.992	0.984	0.950	0.884	0.911	0.862	0.949	0.948	0.950
B‐NHL subtypes differentiation	CD30, CD5, EBERs, CD20, CD10, TdT, CD23, CyD1	Internal validation	0.940	0.853	0.681	0.478	0.491	0.485	0.664	0.658	0.681
		Temporally independent validation	0.950	0.884	0.662	0.489	0.585	0.472	0.646	0.666	0.662
		External validation	0.912	0.869	0.636	0.467	0.487	0.489	0.642	0.691	0.636
DLBCL subtypes differentiation	CD10, MUM‐1	Internal validation	0.965	0.960	0.963	0.961	0.964	0.958	0.963	0.963	0.963
		Temporally independent validation	0.954	0.948	0.943	0.938	0.936	0.941	0.943	0.943	0.943
		External validation	0.973	0.962	0.931	0.921	0.908	0.941	0.933	0.939	0.931
T‐NHL subtypes differentiation	CD30, CD10, EBERs, CD56,TIA1,CD5, TdT, ALK, CXCL‐13	Internal validation	0.976	0.971	0.857	0.857	0.858	0.860	0.860	0.868	0.857
		Temporally independent validation	0.951	0.959	0.847	0.811	0.819	0.808	0.848	0.856	0.847
		External validation	0.952	0.955	0.842	0.799	0.815	0.788	0.840	0.841	0.842

Abbreviations: DLCBL, diffuse large B‐cell lymphoma; HL, Hodgkin lymphoma; NHL, non‐Hodgkin lymphoma.

**TABLE 3:** Comparison between expected panel size in proposed system and actual panel size in clinical settings.

		The internal primary‐stage cohort (recorded January 2015–June 2019 at Sun Yat‐sen University Cancer Center, *N* = 4263)					The internal later‐stage cohort (recorded July 2019–August 2021 at Sun Yat‐sen University Cancer Center, *N* = 4545)					The external cohort (recorded January 2015–December 2019 at Peking University Cancer Hospital & Institute, *N* = 6119)				
Task of classification	Subtypes* final leaf	Counts of records	Number of markers measured in the clinical setting	Cumulative number of markers required by the system	Saving (%)	*p* value	Counts of records	Number of markers measured in the clinical setting	Cumulative number of markers required by the system	Saving (%)	*p* value	Counts of records	Number of markers measured in the clinical setting	Cumulative number of markers required by the system	Saving (%)	*p* value
HL and NHL differentiation	* HL	347	13.58	4	70.54%	<0.001	345	13.65	4	70.70%	<0.001	690	9.96	4	59.84%	<0.001
	NHL															
NHL subtypes differentiation	B‐NHL		12.35	9	27.13%	<0.001		13.59	9	33.77%	<0.001		9.59	9	6.15%	0.003
	T‐NHL															
	* NK‐NHL	343					329					293				
B‐NHL subtypes differentiation	DLBCL		12.42	12	3.38%	0.001		12.78	12	6.10%	<0.001		10.92	12	−9.89%	<0.001
	* FL	449					399					797				
	* MZL	451					524					529				
	* MCL	126					119					242				
	* BL	151					88					48				
	* SLL	86					62					81				
	* PMLBCL	52					34					57				
	* B‐LBL/ALL	38					31					31				
	* Others	35					27					20				
DLBCL subtypes differentiation	* Non‐GCB	519	14.94	13	12.99%	<0.001	390	15.59	13	16.61%	<0.001	728	12.6	13	−3.17%	<0.001
	* GCB	327					228					314				
T‐NHL subtypes differentiation	* ALCL	132	13.5	12	11.11%	<0.001	129	15.98	12	24.91%	<0.001	140	11.76	12	−2.04%	0.21
	* AITL	102					131					176				
	* TLBL	137					93					134				
	* PTCL‐U	77					50					69				
	Average number of markers to reach the final leaves		**13.31**	**11.12**	**16.45%**	**<0.001**		**13.99**	**10.95**	**21.73%**	**<0.001**		**11.18**	**10.77**	**3.67%**	**<0.001**

Abbreviation: AITL, angioimmunoblastic T‐cell lymphoma; ALCL, anaplastic large cell lymphoma; BL, Burkitt lymphoma; B‐LBL/ALL, B lymphoblastic lymphoma/leukemia; DLCBL, diffuse large B‐cell lymphoma; FL, follicular lymphoma; GCB, germinal center B cells; HL, Hodgkin lymphoma; MCL, mantle cell lymphoma; MZL, marginal zone lymphoma; NHL, non‐Hodgkin lymphoma; Others, other B‐NHL subtypes; PMLBCL, primary mediastinal (diffuse) large B‐cell lymphoma; PTCL‐U, peripheral T‐cell lymphoma‐unspecified; SLL, small lymphocytic lymphoma; TLBL, T lymphoblastic lymphoma.

Bold values denote statistical significance at the *p* < 0.001 level.

We first evaluated AUCs using simplified panels in the three validation sets. For the five tasks above, in the internal validation set, AUCs (percentages of corresponding full model) results were 0.961 (100.08% of AUC in full model), 0.980 (98.71%), 0.853 (90.67%), 0.971 (99.45%), and 0.960 (99.50%); in the temporally independent validation set, results were 0.956 (99.99%), 0.983 (99.00%), 0.884 (92.99%), 0.959 (100.84%), and 0.948 (99.35%); in the external validation set, results were 0.961(100.10%), 0.984 (99.22%), 0.869 (95.27%), 0.955 (100.37%), and 0.962 (98.91%).

We also compared the number of markers required in our approach to the number of makers actually measured in real‐world clinical scenarios. The hierarchical classification system required an average of 11.12, 10.95, and 10.77 markers to reach the final leaf of subtypes in the internal primary‐stage, internal later‐stage, and external cohorts, respectively. In contrast, the numbers of markers measured in clinical settings were significantly higher, at 13.31, 13.99, and 11.18 in the internal primary‐stage, internal later‐stage, and external cohorts, respectively. Our findings suggest that the hierarchical classification system has the potential to reduce the number of markers required in clinical settings in the future.

## 4 DISCUSSION

This study identified a simplified panel of markers for each task of lymphoma subtype categorization using a machine learning approach. As a result, a hierarchical classification approach for determining lymphoma pathological subtypes using EBER in situ hybridization and IHC markers was generated and independently validated. The design of the system was consistent with the real situation in clinical settings in terms of both workflow and data validity. First, the hierarchy architecture mimics the workflow of identifying lymphoma subtypes in the real scenarios. Second, pathologists have unique marker needs to fulfill these tasks, which were represented in the filtered datasets. Moreover, compared to an all‐in‐one classifier, the stratification of each level of the hierarchy into independent tasks aided the machine learning algorithm's model fitting.

Machine learning has been introduced as an effective classification approach for lymphoma subtypes.^6^, ^7^, ^8^, ^9^, ^10^, ^11^ DT was the first machine learning method to validate cell‐of‐origin (COO) DLBCL classification based on IHC data,^6^, ^7^ and non‐linear algorithms such as SVM and MLP have also been effectively applied to the classification of various tumors.^12^, ^13^ SVM demonstrates remarkable proficiency in discerning intricate patterns that may signify distinct cancer subtypes within complex datasets. Its ability to model complex, non‐linear boundaries makes it a potent tool for classifying data with subtle distinctions. As a fundamental type of artificial neural network, MLP confers significant advantages in the realm of complex pattern recognition. Its layered structure facilitates the capture of nuanced relationships within medical data, thereby enhancing diagnostic accuracy. RF and xGBoost, ensemble methods that build upon decision trees, have also garnered extensive validation for its efficacy in classification tasks. Therefore, in the present study, we applied DT, SVM, and MLP in addition to LR, RF, and xGBoost to the five classification tasks using the complete panel of markers, and LR outperformed other models after validation. Although IHC DT algorithms currently provide convincing results for DLBCL classification, our results demonstrated that LR was superior to DT in terms of discriminatory ability. In addition, the results revealed that lymphoma subtypes may be classified using a linear combination of related markers with varying weights.

It was noticed that the classification of B‐cell lymphoma subtypes was the task with the lowest average AUC across the three validation sets whichever algorithm implemented. This is because certain B‐cell lymphoma variants are frequently difficult to distinguish merely on basis of immunostaining,^14^, ^15^ and they can be identified more accurately by combining IHC data with clinical and pathological variables. Lymphoma differential diagnosis may benefit in the future by the consolidation of information from numerous sources.

Although the system based on models derived from full panels of markers had good performance, it required a total number of 32 distinct markers, which can be costly in clinical settings. The panel size could be lowered in two ways. First, machine learning approaches can simplify the panels of markers by determining the most crucial markers for each task. Second, hierarchical decision flow architecture enabled the re‐use of previously tested markers in the upper level, so simplifying the panels even further. For instance, in the differentiation of NHL subtypes, our framework utilizes CD30 and CD5, which were initially part of the full marker panel and have been incorporated at higher levels of the system's architecture (as shown in Figure 2A). To augment the diagnostic capabilities, we then further added essential markers. In this particular task (depicted in Figure 2B), CD20‐a universally recognized pan‐B‐cell marker that is expressed throughout various stages of B‐cell development‐has been incorporated as a key addition.

We noticed that for some tasks of classification, the proposed approach required more markers than those actually measured in the external validation cohort. As we further investigated, the possible reason is that pathologists at Peking University Cancer Hospital & Institute integrated IHC results from other hospitals where participants were treated previously for diagnosis. However, these part of IHC markers were not recorded in the system where we extracted data, and thus, were not counted.

Our study identified and verified simplified panels of markers for the system based on LR algorithm, which showed to be cost‐effective, efficient, and connected to previous work. First, the required panels of markers were consistent with earlier findings. For example, our system's classification results for COO subtypes of DLBCL agreed with the Hans algorithm,^7^, ^8^ without the use of Bcl‐6. Our system has identified CD10 and MUM‐1 for the assignment, which produced the same results as the Hans algorithm. Second, our suggested approach required fewer markers compared to earlier investigations. Earlier DLBCL classification studies, for instance, mostly utilized decision tree algorithm and evaluated two to five antibodies, such as CD10, Bcl‐6, FOXP1, GCET1, and MUM‐1.^6^, ^7^, ^8^, ^9^, ^16^ However, our system required only CD10 and MUM‐1 for the subtype categorization of DLBCL. Additionally, our method required much fewer indicators than those evaluated in the three cohort sets from real‐world clinical contexts. Lastly, we tested the performance of our system using simplified panels of markers in the three validation sets, and the results indicated that the AUCs in most tasks increased or were at least 99% (95% in the classification of B‐cell lymphoma subtypes) of the corresponding full models. This suggested that the simplified panels assisted in preventing overfitting. Taken together, our results suggest that it is possible to minimize the number of immunohistochemical testing items while preserving discriminative capacity, hence saving money.

The simplified panel of markers and hierarchy proposed in this study have the potential to benefit both clinical practice and research field. First, this approach could help pathologists with panel selection and differential diagnosis by reducing the lymphoma diagnostic workflow. Second, this system is interpretable, as the coefficients of markers permit comprehension of LR‐based model. Lastly, the newly identified relevant markers, which were not regarded as minimum requirement by pathologists, may provide valuable hints for future investigations and serve as viable options for lymphoma differential diagnosis.

Our investigation has several limitations that should be noted. First, both the internal and external cohorts were exclusively Chinese; therefore, it is uncertain whether our findings can be applied to other ethnic groups. However, both centers where the data was collected are significant national cancer centers located in various regions of China, which may boost the population‐representativeness of the cohorts. Second, the high proportion of missing data across all datasets may add inaccuracies into our findings. Nevertheless, our findings can be enhanced by the vast quantity of lymphoma data and independent validation from outside sources. Third, the use of EBER in situ hybridization and IHC markers alone as predictors in our study, while appropriate for certain tasks such as DLBCL subtype classification, may not be optimal for other tasks such as B‐cell lymphoma subtype classification, where additional information such as morphological, molecular, and clinical features, as well as viral and immune status could be useful. Last but not least, as this system was designed for a clinical setting where preliminary classification has already been made, it was not suited for environment that requires comprehensive differential diagnosis.

In this multicenter study of 14,927 patients, we developed and validated a hierarchical classification approach for the differential diagnosis of lymphoma using simplified marker panels. The system demonstrated promising results in both temporal independent and local independent datasets. The potential application of this approach in lymphoma may assist pathologists in panel selection and streamline the workflow, thus potentially saving medical expenses in future clinical practice.

## AUTHOR CONTRIBUTIONS

**Jiming Xu:** Data curation (equal); formal analysis (equal); investigation (equal); methodology (equal); visualization (equal); writing – original draft (equal). **Yunfei Shi:** Data curation (equal); formal analysis (equal); investigation (equal); methodology (equal); project administration (equal); visualization (equal); writing – original draft (equal). **Mengxuan Cui:** Data curation (supporting); formal analysis (supporting); investigation (supporting); methodology (supporting); visualization (equal); writing – original draft (equal). **Yao Wang:** Data curation (supporting); formal analysis (supporting); investigation (equal); methodology (supporting); visualization (equal); writing – original draft (equal). **Wenhui Fan:** Writing – review and editing (supporting). **Jingping Yun:** Writing – review and editing (supporting). **Linfeng Li:** Conceptualization (equal); project administration (equal); supervision (equal); writing – review and editing (equal). **Muyan Cai:** Conceptualization (equal); funding acquisition (lead); project administration (equal); supervision (equal); writing – review and editing (equal).

## ETHICS STATEMENT

This study was approved by the ethical review committee of the Peking University Cancer Hospital & Institute and Sun Yat‐sen University Cancer Center. The ethical review committee waived the requirement for informed consent due to the retrospective nature of the study.

## Supporting information


Appendix S1:

## Data Availability

Data available: YesData types: Deidentified participant data, Data dictionaryHow to access data: Zenodo. https://doi.org/10.5281/zenodo.11366453When available: With publicationSupporting DocumentsDocument types: NoneAdditional InformationWho can access the data: Scientific researchersTypes of analyses: For scientific researching purposeMechanisms of data availability: Public.
