# Supplementary material for: Development and validation of a hierarchical approach for lymphoma classification using immunohistochemical markers
Source: Cancer Med. 2024 Oct 23;13(20):e70120. doi: 10.1002/cam4.70120 (PMC11499568; doi:10.1002/cam4.70120)
Supplement: Supplementary file 1 — Appendix S1: [file CAM4-13-e70120-s001.docx]

**SUPPLEMENT 1.**

**eMethods.**

**eFigure 1.** The 4^th^ revised edition of the WHO classification of Hematopoietic Tumors

**eFigure 2.** A classical Hodgkin Lymphoma case with HRS cells


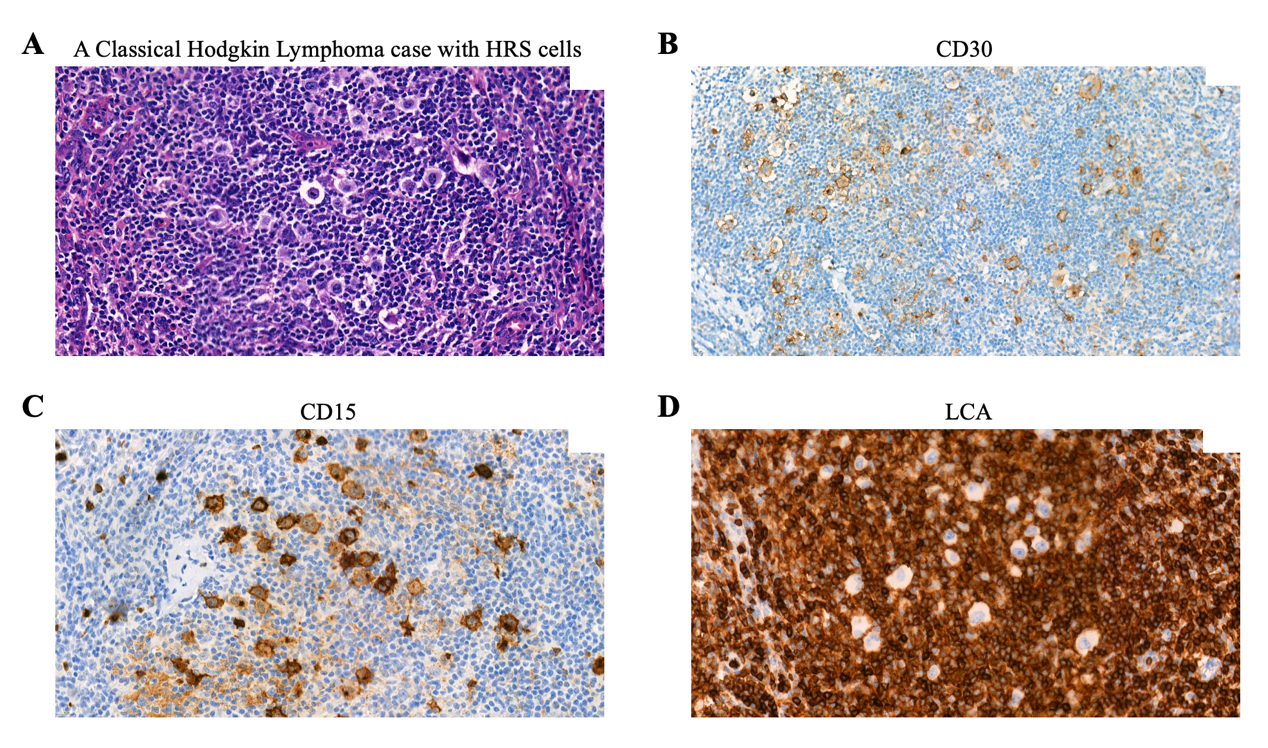


**eFigure 3.** A case diagnosed as Burkitt lymphoma with medium sized B cells


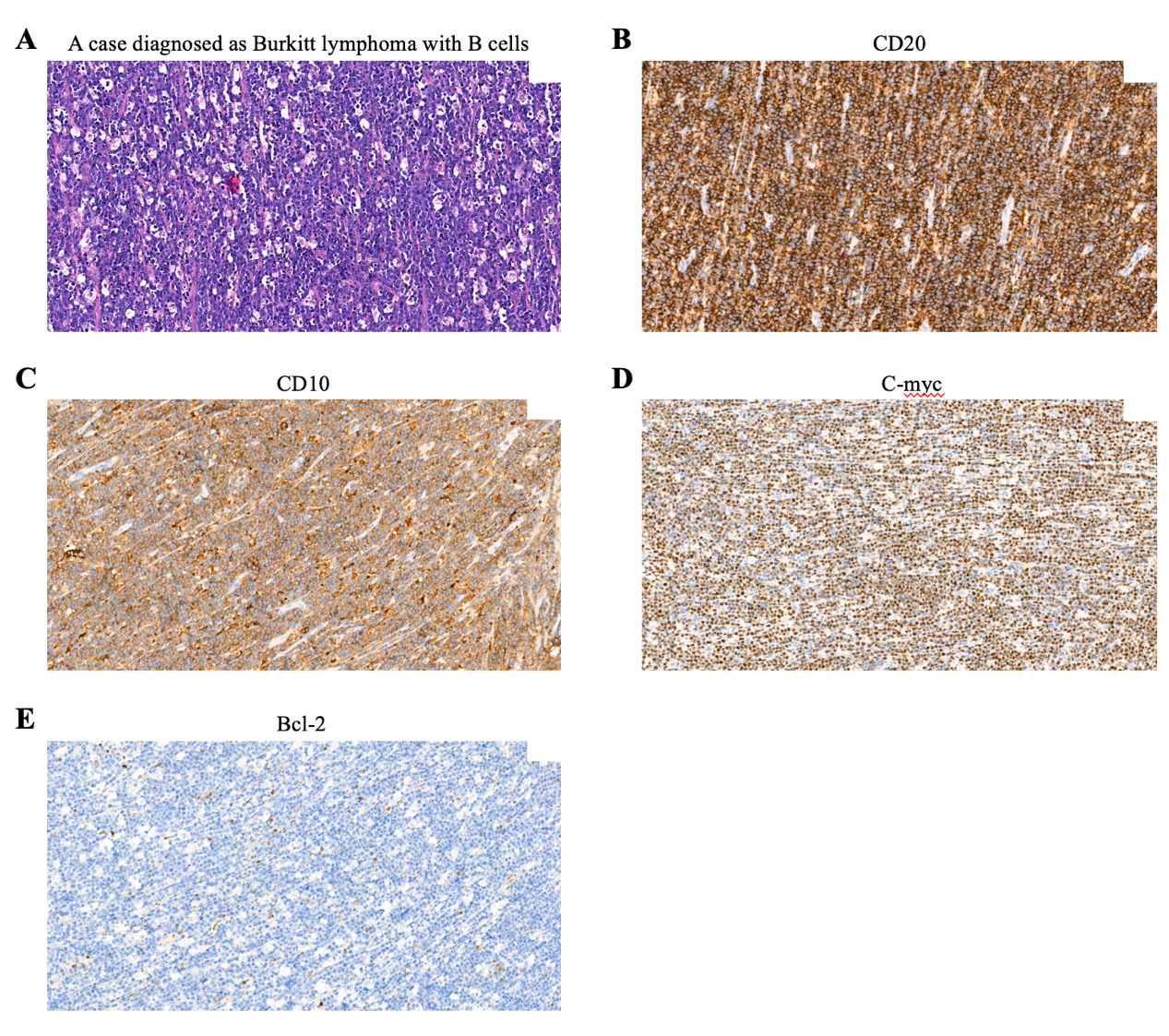


**eFigure 4.** A case of extra nodal NK/T cell lymphoma with medium sized atypical tumor cells


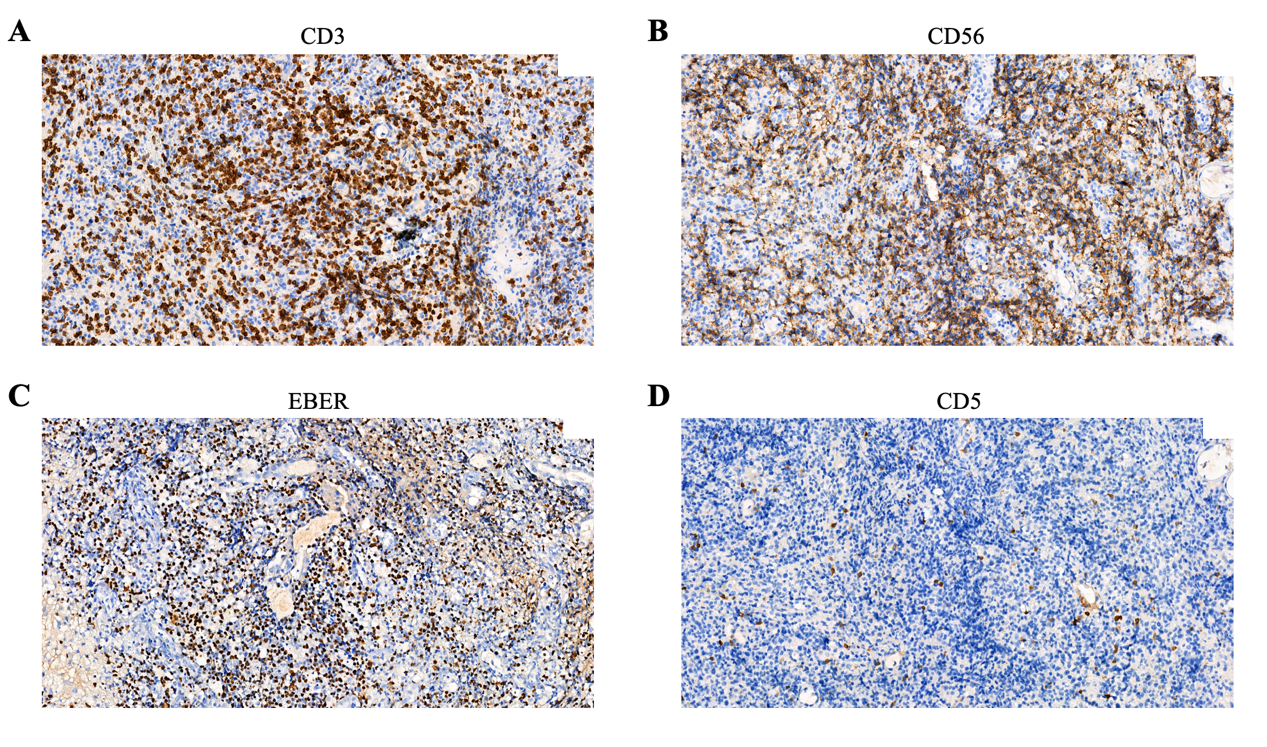


**eFigure 5.** A case diagnosed as angioimmunoblastic T cell lymphoma with diffusely infiltrating medium sized lymphoid tumors cells


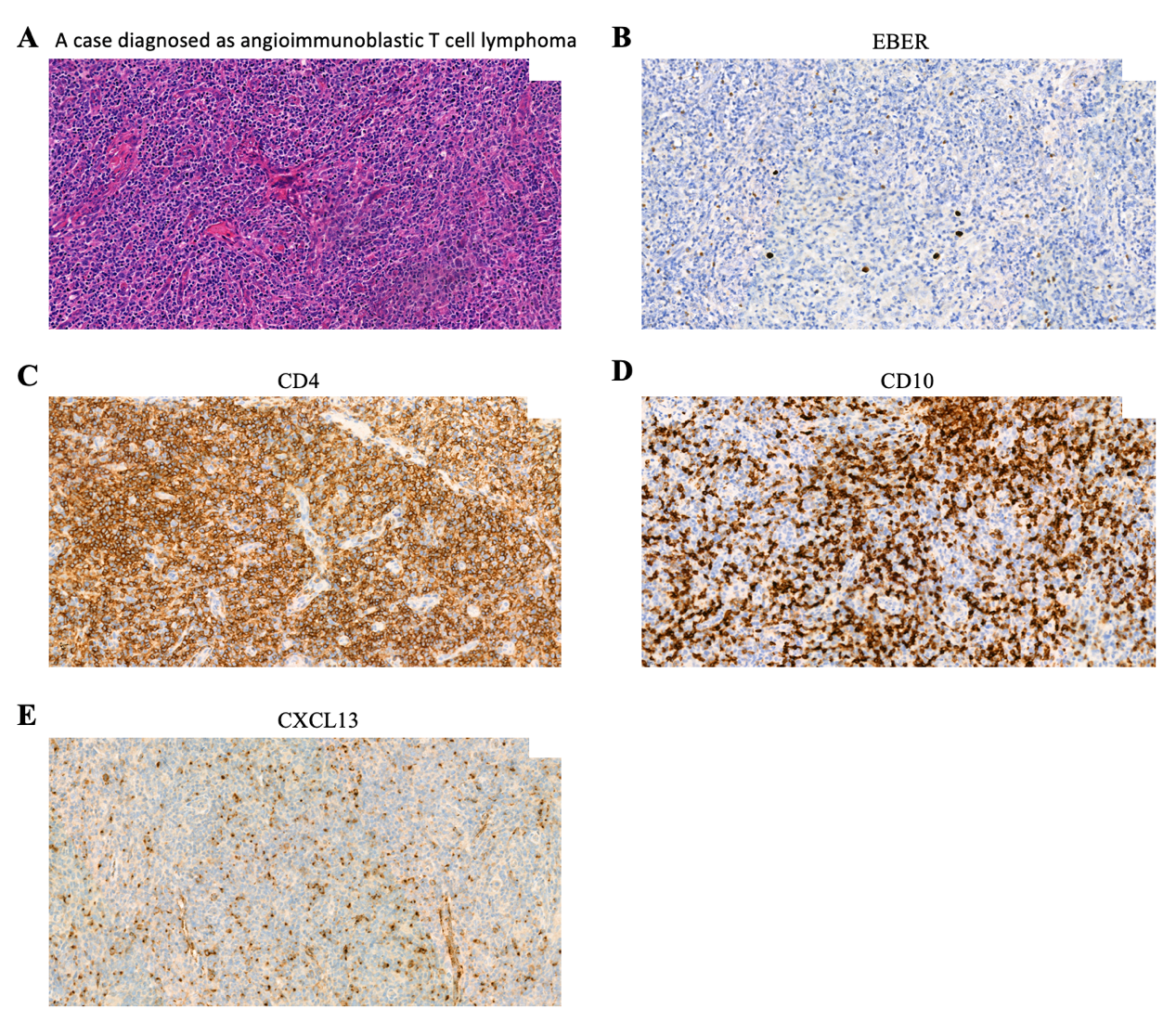


**eResults**

**eTable 1.** EBER in situ hybridization and IHC markers characteristics of the train set

**eTable 2.** EBER in situ hybridization and IHC markers characteristics of the internal validation cohort

**eTable 3.** EBER in situ hybridization and IHC markers characteristics of the temporal independent validation cohort

**eTable 4.** EBER in situ hybridization and IHC markers characteristics of the external validation cohort

**eTable 5.** AUCs of the six machine learning models in the four sets

**eTable 6.** Coefficients of markers in the *Full Models*, shown in descending order of feature importance (measured by absolute values of coefficients)

**eTable 7.** Coefficients and intercepts of markers in the *Simplified Model*s

**SUPPLEMENT 2. Data Sharing Statement**

Data are available by reasonable request ([caimy@sysucc.org.cn](mailto:caimy@sysucc.org.cn)) or https://doi.org/10.5281/zenodo.11366453.
